# THE IMPACT OF PARTICIPATION IN AN IMMERSIVE VIRTUAL REALITY TAI CHI PROGRAM ON THE BALANCE OF OLDER ADULTS

**DOI:** 10.1093/geroni/igae098.4089

**Published:** 2024-12-31

**Authors:** Jaehyun Kim, Marcos Ardon Lobos, Serim Park, Seungjun Oh, Jay Kim, Junhyoung Kim

**Affiliations:** East Carolina University, Greenville, North Carolina, United States; East Carolina University, Greenville, North Carolina, United States; Texas A&M University, College Station, Texas, United States; Chadwick International School, Incheon, Republic of Korea; Texas A&M University, College Station, Texas, United States; Texas A&M University, College Station, Texas, United States

## Abstract

Falls are the leading cause of injury-related mortality in older adults aged over 65 years, with ~36 million older adults suffering from falls each year that result in more than 34,000 deaths. Tai Chi, an ancient Chinese martial art, is shown to reduce the risk of falls by improving physical functioning (e.g., balance, flexibility, muscle strength). Despite the well-recognized health benefits of Tai Chi instruction, older adults often encounter barriers including limited access to programs, a lack of instructors, and environmental distractions. To address these barriers, this study examined the impact of an immersive virtual reality Tai Chi intervention (IVTC) on the balance of older adults. Eighteen older adults from senior centers in North Carolina participated in eight 15-minute IVTC sessions over a month and completed the Berg Balance (BB) test before and after the IVTC intervention program. The results showed significant improvement in BB scale scores after the IVTC sessions (p <.001) with a large effect size (d = -2.75). Holden’s System Usability Scale indicated that participants were very favorable towards using the program. Our findings suggest that the IVTC program is a very usable intervention that could effectively improve the balance of older adults, highlighting its potential as an innovative intervention to reduce fall risks for older adults.

